# NO Network for Plant–Microbe Communication Underground: A Review

**DOI:** 10.3389/fpls.2021.658679

**Published:** 2021-03-17

**Authors:** Anjali Pande, Bong-Gyu Mun, Da-Sol Lee, Murtaza Khan, Geun-Mo Lee, Adil Hussain, Byung-Wook Yun

**Affiliations:** ^1^Laboratory of Plant Molecular Pathology and Functional Genomics, Department of Plant Biosciences, School of Applied Biosciences, College of Agriculture and Life Science, Kyungpook National University, Daegu, South Korea; ^2^Department of Entomology, Abdul Wali Khan University, Mardan, Pakistan

**Keywords:** rhizosphere, microbes, nitrogen fixation, silicon, signaling, nitric oxide

## Abstract

Mechanisms governing plant–microbe interaction in the rhizosphere attracted a lot of investigative attention in the last decade. The rhizosphere is not simply a source of nutrients and support for the plants; it is rather an ecosystem teeming with diverse flora and fauna including different groups of microbes that are useful as well as harmful for the plants. Plant–microbe interaction occurs *via* a highly complex communication network that involves sophisticated machinery for the recognition of friend and foe at both sides. On the other hand, nitric oxide (NO) is a key, signaling molecule involved in plant development and defense. Studies on legume–rhizobia symbiosis suggest the involvement of NO during recognition, root hair curling, development of infection threads, nodule development, and nodule senescence. A similar role of NO is also suggested in the case of plant interaction with the mycorrhizal fungi. Another, insight into the plant–microbe interaction in the rhizosphere comes from the recognition of pathogen-associated molecular patterns (PAMPs)/microbe-associated molecular patterns (MAMPs) by the host plant and thereby NO-mediated activation of the defense signaling cascade. Thus, NO plays a major role in mediating the communication between plants and microbes in the rhizosphere. Interestingly, reports suggesting the role of silicon in increasing the number of nodules, enhancing nitrogen fixation, and also the combined effect of silicon and NO may indicate a possibility of their interaction in mediating microbial communication underground. However, the exact role of NO in mediating plant–microbe interaction remains elusive. Therefore, understanding the role of NO in underground plant physiology is very important, especially in relation to the plant’s interaction with the rhizospheric microbiome. This will help devise new strategies for protection against phytopathogens and enhancing plant productivity by promoting symbiotic interaction. This review focuses on the role of NO in plant–microbe communication underground.

## Introduction

Plants develop a close association with the microorganisms inhabiting the areas in and around the roots which are influenced by the chemicals (and/or enzymes) released from the plant roots. The collective term for microbial communities in the rhizosphere is the root microbiome. The rhizosphere harbors the largest reservoir of microbial diversity on earth. Several beneficial microbes are not only able to suppress diseases in host plants by inhibiting the growth of soil-borne phytopathogens, but they also stimulate growth and defense responses in host plants by the production of growth regulators, phytohormones, and other substances like antibiotics, antifungal metabolites, and defense enzymes for pathogen control and trigger induced systemic resistance (ISR) *via* methyl jasmonate and methyl salicylate in plants, displaying a sophisticated signaling network through which they communicate. Signaling generally involves signal perception through the cell wall or cell membrane receptors, ion channels and sensors, signal transduction through secondary messengers including cyclic nucleotides, lipid messengers, ions, free radicals, and gases like nitric oxide (NO). These secondary messengers then activate target-responsive genes and stimulate metabolic and physiological responses (Yamaguchi-Shinozaki and Shinozaki, 2006; Pérez-Clemente et al., 2013). Particularly, in the rhizosphere, there exists a multitude of microbial processes where NO is involved. In plants, two isoforms of the nitrate reductase (NR) enzyme catalyze the production of NO from nitrate, though multiple other pathways for NO production also exist. However, being highly reactive NO may prove toxic to the plants. It, therefore, needs to be converted to a non-toxic and ubiquitous form. This is accomplished by the covalent attachment of NO group (–NO) to exposed cysteine thiols to form S-nitrosothiols (SNOs), a process termed S-nitrosation which is a highly ubiquitous post-translational modification of proteins with diverse regulatory roles across the different kingdoms of life. These SNOs serve as mobile reservoirs, providing a sustained supply of NO *in vivo*, playing key roles in plant physiology under basal as well as stress conditions. At the cellular level, NO reacts with glutathione (GSH) to form S-nitrosoglutathione (GSNO) a major cellular reservoir of NO, capable of transferring NO bioactivity to other proteins. The Cellular GSNO levels are controlled by an evolutionarily conserved cytosolic enzyme GSNO reductase 1 (GSNOR1).

At the cellular level, biotic and abiotic stress responses are characterized by immediate redox bursts potentiated by reactive oxygen and reactive nitrogen species. Decades of research reveal an intricate balance between various ROS and RNS to maintain a physiological equilibrium under stress. The magnitude, intensity, and other characteristics of RNS-mediated redox bursts vary depending upon the type, magnitude, and intensity of the stress. The role of NO in plant–pathogen interaction has been studied. However, a lot is still unknown, about the different roles NO plays during plant–microbe interactions. Information about the role of NO during the plant’s interaction with useful microorganisms in the rhizosphere such as the rhizobacteria, mycorrhizae, free-living plant growth-promoting rhizobacteria and fungi, and others is specifically scarce. In this review, we discuss the role of NO in plant–microbe interaction in the rhizosphere.

## Role of NO in Root Development

The role of NO in morphological growth and developmental pattern of root and other developmental processes like photomorphogenesis, senescence, and leaf expansion in many plant has already been elucidated (Beligni and Lamattina, 2001; Kwon et al., 2012; Baudouin and Hancock, 2014). Also, reports of its role are suggested in rapid physiological responses like stomatal closure (Desikan et al., 2004) and the cytokinin signaling pathway (Tun et al., 2001). However, its role in influencing root growth and developmental processes via auxin-regulated signaling cascade is considered as one of the most important functions in plant biology. The role of NO in root development has been thoroughly reviewed by Molina-Favero et al. (2007). NO influences auxin signaling through S-nitrosation of the auxin receptor TIR1 at cys140 (Terrile et al., 2012). The view that NO is involved in root development also seems to have some connections with the root microbiome. Therefore, we aimed to review the studies that could suggest links between NO role in root development and its links with the root microbiome.

Several studies suggest the involvement of auxin-induced NO-dependent pathway during adventitious root formation (Pagnussat et al., 2004; Lanteri et al., 2006). Pagnussat et al. (2002) reported the first study suggesting that auxin induces adventitious root formation through an increase of the NO concentration at the base of cucumber hypocotyls. Soon after this study, the same group demonstrated that auxin and NO trigger both cGMP-dependent and cGMP-independent pathways leading to adventitious root formation. Moreover, the role of NO in promoting root elongation has also been reported in maize plants (Gouvea et al., 1997). Other studies also support the involvement of NO in phosphorous deficiency-induced lateral root development and growth in *Lupinus albus* (Wang et al., 2010; Meng et al., 2012), NO-induced NADPH oxidase in adventitious root growth in *Panax ginseng* (Tewari et al., 2008; Tewari and Paek, 2014), *Azospirillum brasilense*-induced NO in lateral root formation in tomato (Creus et al., 2005), and NO-induced modulation in adventitious root growth in *Vigna radiata* (Sharma et al., 2019) and rice (Kushwaha et al., 2019). Furthermore, a recent study also suggests that NO is involved in the brassinolide-induced adventitious root development in cucumber (Li et al., 2020). Root hair initiation and elongation in lettuce also involves NO (Lombardo et al., 2006) and is critical in determining root hair differentiation and elongation, mediating an auxin-triggered signaling cascade. Similar regulation by NO and auxin has also been reported in *Arabidopsis* (Lombardo et al., 2006). Interestingly, bacterial quorum-sensing molecules are also reported to have a role in plant defense responses and root development. For instance, molecules like *N*-3-oxo-decanoyl-L-homoserine-lactone can induce adventitious root formation through the activation of cGMP signaling in a hydrogen peroxide and NO-dependent manner in mung bean (Bai et al., 2012). Another study has also found enhanced plant growth and root development accelerated by arbuscular mycorrhizal fungi in trifoliate orange (Tian et al., 2017). Furthermore, the role of NO in root architecture has been recently reviewed by Mukherjee and Corpas (2020).

## NO in Plants and Bacteria

In plants, NO is produced through enzymatic and non-enzymatic reactions. One of the enzymatic reactions involves L-arginine as a primary substrate for NO synthase (NOS) leading to the production of NO through oxidative pathway (Gupta et al., 2011). The first report of putative NOS activity in plants was from the roots and nodules of *L. albus via* indirect detection of NOS antagonist N(G)-monomethyl-L-arginine, inhibiting the production of L-citrulline (Cueto et al., 1996). A unique *AtNOS1* gene was isolated from *Arabidopsis thaliana* and its mutant (*Atnos1*) showed lesser NO content as compared to the wild type (Guo et al., 2003). Later on, it was renamed NO-associated protein 1 (AtNOA1) due to its inability to bind and oxidize arginine (Moreau et al., 2008). In 2010, a study described the presence of NOS enzyme in *Ostreococcus tauri*, a single-celled alga with mostly those characteristics that are present in animal NOS, thus suggesting the presence of NOS in plants (Foresi et al., 2010). However, until now the production of NO via a canonical NOS enzyme remains controversial (Hancock, 2020). NO production through enzymatic reaction also occurs through NR which catalyzes the conversion of nitrate and nitrite to NO (Tejada-Jimenez et al., 2019). This process is well established for NO production in several plant species including maize, cucumber, spinach, and sunflower (reviewed in detail by Cohen et al., 2009).

In the case of bacteria, the reduction of nitrite to NO is catalyzed by nitrite reductase which acts as a key enzyme in the dissimilative denitrification chain. Dissimilative nitrite reductase is therefore considered the major known source of NO in bacteria. Two distinct classes of dissimilatory nitrite reductases may contain either copper or heme as a cofactor, out of which heme-containing enzyme is more frequent. The biological relevance of this redundancy is only speculated to be associated with the availability of copper and iron in different microenvironments because enzymes containing copper and heme never coexist within the same bacterial species (Cutruzzolà, 1999). Moreover, bacterial NOS (bNOS) activity was first reported in the genus *Nocardia* (Chen and Rosazza, 1994). Later, it was reported in many plant-associated and free-living bacteria including *Sinorhizobium, Mycobacterium, Nocardia, Rhodococcus, Streptomyces, Bacillus, Geobacillus*, and *Paenibacillus* by Cohen et al. (2009). Since bacteria and plants share several similar features of NO-producing pathways, NO is suggested to act as a signaling molecule in plant–microbe interaction.

Common NO-mediated signaling links in plants and microbes have also been reported in a few studies. An important role of NO in plants is its influence on cellular processes through protein S-nitrosylation. Interestingly, endogenous S-nitrosylation is also reported to occur in *Escherichia coli* as a prominent feature of anerobic respiration which is regulated by OxyR, a regulon that controls endogenous S-nitrosylation in *E. coli* (Seth et al., 2012). Studies have also suggested the involvement of OxyR in influencing virulence of plant pathogens (Flores-Cruz and Allen, 2011; Ishiga and Ichinose, 2016).

## Plant–Microbe Interaction: NO and the Plant Innate Immune System

Several plant associate microbes are pathogens that impair growth and reproduction. As such, there would hardly be any defense without the pathogen. Hence, the co-evolution of plant–microbe interaction shapes the defense system of both plants and microbes over time. Jones and Dangl (2006) proposed that plants possess a two-branched innate immune system that responds to infection. The first branch which is recognized by a set of receptors, also known as pattern-recognition receptors (PRRs), recognizes conserved microbe-associated molecular patterns (MAMPs). Typical examples of these receptors are LRR receptor-like kinases including *Arabidopsis* FLAGELLIN-SENSING 2 (FLS2) which recognize a peptide sequence (22 amino acids) present in bacterial flagellin and EF-Tu receptor (EFR), recognizing active epitope of bacterial elongation factor. Therefore, these receptors and the microbial molecules recognized by them constitute an essential part of defense related to innate immunity. On the other hand, the second branch comprises large families of intracellular immune receptors that encode NBS-LRR (nucleotide-binding site leucine-rich repeat) proteins. Meyers et al. (2003) reported the presence of 140 NBS-LLR encoding proteins from the Arabidopsis genome, whereas the japonica rice genome encodes 500 NBS-LRR proteins (Zhou et al., 2004). However, PRRs activate their specific immune response through pathogen-triggered immunity (PTI), and the NBS-LRRs activate it through effector-triggered immunity. The activation of downstream innate immune response through both these branches initiates with changes in the cytoplasmic Ca^2+^ levels, production of reactive oxygen species, and post translational activation of mitogen-activated protein kinase (MAPK) cascade (Jonak et al., 2002). Furthermore, NO is produced when plants are treated with MAMPs or during fungal infection which indicates an important role of NO in activating the innate defense response (Clarke et al., 2000). In fact, literature reports the production of NO during the establishment of interaction between nominally compatible plants and bacteria (Conrath et al., 2004; Mur et al., 2005), and also when plants are treated with lipopolysaccharide (LPS), NO mediates the activation of innate defense genes (Zeidler et al., 2004). Moreover, NO also mediates the regulation of NADPH-generating enzymes through post-translational modifications particularly, tyrosine nitration and S-nitrosation. NADPH is a versatile coenzyme involved in cellular growth, proliferation, detoxification processes, and maintenance of cellular redox status (Corpas and Barroso, 2014).

The important pathways which confer plant immune response are either salicylic acid or jasmonic acid/ethylene pathways. While salicylic acid plays a key role in plant immunity against biotrophic pathogens, it has antagonistic function against the jasmonic acid pathway which is essential role player in providing immunity against necrotrophic pathogens and also during physical wounding (Glazebrook, 2005). Along each signaling pathway, NO can either induce or suppress signaling. It initiates salicylic acid biosynthesis and enables nitrosylation of key cysteines on TGA-class transcription factors to initiate salicylic acid-dependent gene expression. During pathogenic attack, salicylic acid concentration increases in response to the changes in the cellular redox state. This leads to the reduction of disulfide bonds of NPR1 oligomer releasing monomers which translocate to the nucleus activating pathogenesis-related (PR) genes. However, oligomerization of NPR1 occurs under higher levels of SNO *via* S-nitrosylation. This was shown in *Arabidopsis* mutant *atgsnor1-3* where NPR1 oligomer accumulates due to increased GSNO levels which enabled S-nitrosylation at cysteine (Cys)-156, while reducing monomerization by salicylic acid thus resulting in delayed activation of PR genes and enhanced pathogen susceptibility (Tada et al., 2008). Furthermore, Mur et al. (2013) have reviewed the salicylic acid and jasmonic acid/ethylene pathways mediated by NO during plant defense response in much detail. However, investigation is required to understand how this signaling network is mediated and controlled during plant–pathogen interaction in the rhizosphere.

## Signaling in Plant–Microbe Interaction

Some mechanism of recognition or exchange of molecular signals between plants and microbes must be present which determines the specificity of host–microbe interactions. Certainly, for plant–microbe interaction to occur, there must be exchange of some signaling molecules, which is necessary for interaction among them. The initiation of plant–microbe interaction generally involves signal exchange through specific molecules produced by host or microbe or both during recognition which leads to the biochemical, physiological, and molecular responses that affects development of their interaction. Signals initiating, maintaining, and controlling the interaction determine the process of successful interactions. Only the compatible microbes can alter the host defense response out of the millions of microbes present in the rhizosphere and their communication affects plant health and growth. Therefore, the focus of recent research is to determine the signals and regulatory networks which counteract immune detection and recognize beneficial/compatible rhizospheric microbes. Here, we are focusing on the plant growth-promoting microbes and the various suggested mechanisms through which they interact with the plants. Plant growth-promoting microbes are the beneficial soil microbes (bacteria or fungi) colonizing the plant roots and useful in promoting plant growth and health (Spaepen et al., 2009). They are well known for their role and application in agriculture (Reddy et al., 2014).

The plant–microbe interaction occurs in a highly sophisticated manner, and several specialized metabolites and exudates control the process. As a result, gene expression is altered in either plant or microbe or both the interacting partners leading to either “plant growth, inhibition of soil pathogens, nutrient availability, biofilm development, or accumulation of soil microbes” (Li et al., 2013; Mommer et al., 2016; Sasse et al., 2018). The communication may be inter-/intra-species (microbial quorum sensing (QS) or signaling volatile organic compounds) or inter-kingdom communication (root exudates, exchange of symbiotic signals, bacterial QS signals, and exchange of antimicrobial and volatile organic compounds) (Bukhat et al., 2020). Out of the two, our main focus for this review is the inter-kingdom signaling in the rhizosphere which is widespread, common, and diverse. Microbial association with plants, particularly, symbiotic ones is established through the secretion and emission of beneficial molecules which includes growth regulators like auxins, cytokinins, gibberellins, abscisic acid, salicylic acid, and jasmonic acid and volatile-organic compounds like 2-heptanol, 2-endecanone, and pentadecane (Fahad et al., 2015). These molecules activate the plant transcriptome for specific changes or adjustments.

Some chemical signals are also transmitted from the plant’s side which are sensed by specific microbes for association. Cucumber root exudates are reported to attract *Bacillus amyloliquefaciens* SQR9, while fumaric acid from banana root attracts *Bacillus subtilis* N11; as a result, biofilm formation is reported in both the cases (Zhang et al., 2014). Other root exudates involved in plant–microbe interaction are flavonoids (derivatives of 2-phenyl-1,4-benzopyrone) which trigger nod genes in bacteria to produce lipochitooligosaccharides (LCOs) for initiating nodule formation in the roots of leguminous plants. These LCOs are also reported to participate in the interaction between arbuscular mycorrhizal fungi and plants, mimicking bacterial QS molecules, and playing role in influencing bacterial metabolism (Hassan and Mathesius, 2012). Moreover, plant exudates like aminocyclopropane-1-carboxylic acid (ACC) (Haichar et al., 2012) and tryptophan (Haichar et al., 2014) are also used by rhizobacteria as precursors of growth regulators ethylene and auxin, respectively. A recent study by Ding et al. (2020) suggests that there is alteration in the transcriptome of the plant pathogen, *Fusarium graminearum* which triggers NO production during sensing the presence of host before establishing physical contact with host’s roots. Further, virulence in *F. graminearum* and NO production during host sensing requires FgANK1, a protein containing an ankyrin-repeat domain. FgANK1, which normally resides in the cytoplasm, is translocated to the nucleus in response to the host signal where it interacts with a zinc finger transcription factor (FgZC1) which is also required for the specific binding to the NR promoter, NO production, and virulence in *F. graminearum*. Hence, NO is produced in *F. graminearum* after host is recognized and FgANK1 and FgZC1 regulate it, but still the exact pathway of NO biosynthesis and production in *F. graminearum* or other fungi is still unknown (Ding et al., 2020). Furthermore, one of the studies from our group has also reported the growth promotory effects of *Bacillus aryabhattai* strain SRB02 in soybean which is modulated by the production of phytohormones (Park et al., 2017). Although legume–rhizobia interaction provides much evidence in support of the involvement of NO during plant–microbe interaction, studies need to be conducted in order to understand how NO promotes their interaction.

## Role of NO as Signal Molecule in Plant–Microbe Interaction

Rhizosphere is home to numerous microbes that may or may not affect plant growth, health and productivity directly. However, microbes that do not affect plants directly have several indirect roles to play. The uncertainty about their roles comes from the limited knowledge of the impact that these plant-associated microbes may have on the plants. In-depth studies on rhizospheric symbiotic rhizobia and mycorrhizal fungi has enabled a greater understanding of their association with plants. Moreover, as the review focuses on the role of nitric oxide as an important signaling molecule in mediating the mutual association, our focus is mainly on plant-rhizobium signaling because it is the best understood association where nitric oxide is involved at several steps. Further, this review may draw attention of the researchers involved in studying particular plant-microbe interactions, towards studying the speculated yet unknown role of nitric oxide signaling network involved in mediating the plant-microbe interaction.

Recent research studies have demonstrated some important roles of NO during plant–bacterial interactions (Table 1) (Vaishnav et al., 2018). It has been known that the association of hemoglobins (Hbs) and NO is an important factor between symbiotic interactions. In addition, NO contents in plant and symbiont cells are intimately regulated by Hbs and S-nitrosoglutathione (GSNO) reductase to minimize toxic effects. For pathogen interactions, the expression level of Hb is maintained at low level to perpetuate NO production and promote defense response. In the event of symbiotic interactions, the expression of Hb is rapidly induced and it causes decrease of NO contents and suppression of plant defense system (Hichri et al., 2016). Plant phytoglobins also regulate NO accumulation by functioning as NO dioxygenases, metabolizing NO to nitrate. Studies also suggest its role during plant–microbe interaction in controlling NO bioactivity (Shimoda et al., 2009; Bai et al., 2016). *PHYTOGB1* is reported as one of the early activated during root–mycorrhizal interaction (Hogekamp and Küster, 2013). Furthermore, PHYTOGB1, is also demonstrated to regulate NO levels in tomato roots during arbuscular mycorrhizal symbiosis with R. irregularis in order to promote and control the interaction (Martínez-Medina et al., 2019). Overall, the role(s) of NO in the arbuscular mycorrhizal fungi still remains elusive and its regulation is yet unclear. In arbuscular mycorrhizae interaction, another NO detoxification gene VfLb29 in Vicia faba is also upregulated to repress defense response. Therefore, the best example of the NO as a signaling molecule in plant–microbe interaction is during the process of biological nitrogen fixation. The process of nitrogen fixation is symbiotic association between legume (leguminous plants) and rhizobia (a small group of bacteria) which increases the level of nitrogen in the soil. These rhizobia may be free living or may be associated with epiphytes and endophytes. In some cases, they can also establish endocellular symbiosis with legumes (Masson-Boivin et al., 2009). Thus, the role of NO as a potential signaling molecule during plant–microbe interaction is highlighted in several studies.

**TABLE 1:** Nitric oxide-mediated plant–microbe interaction.

Plant	Bacteria	Function	References
*Medicago truncatula*	*Sinorhizobium meliloti*	Nitrate reductases regulate nitric oxide production and nitrogen-fixing metabolism	Berger et al., 2020
*Triticum aestivum*	*Azospirillum Brasilense*	Lectins of bacterium induced NO in seedlings	Alen’kina et al., 2014
*Arabidopsis thaliana*	*Botrytis cinerea*	NO production mediates oligogalacturonides-triggered immunity and resistance	Rasul et al., 2012
*Medicago truncatula*	*Sinorhizobium meliloti*	Plant NR produced NO present in root hair and nodule primordia	Del Giudice et al., 2011 Horchani et al., 2011
*Arabidopsis thaliana*	*Sclerotinia sclerotiorum*	NO participates in defense-related signaling pathways controlling disease resistance	Perchepied et al., 2010
*Glycine max*	*Bradyrhizobium japonicum*	NO activates bacterial responses to low O_2_ tension in soybean	Leach et al., 2010
*Lotus japonicus*	*Mesorhizobium loti*	Class 1 plant hemoglobin genes enhance symbiotic nitrogen fixation	Shimoda et al., 2009
*Nicotiana benthamiana*	*Botrytis cinerea*	NO participates in disease resistance to necrotrophic pathogen	Asai and Yoshioka, 2009
Tomato	*Pseudomonas fluorescens*	Higher NO production inhibits *Ralstonia solanacearum*	Wang et al., 2005
*Arabidopsis thaliana*	*Pseudomonas syringae*	Nitrite as the major source of nitric oxide during infection	Modolo et al., 2005
Maize	*Pseudomonas fluorescens YT101*	Respiratory nitrate reductase produced NO involved in bacterial colonization	Ghiglione et al., 2000

## NO-Mediated Legume–Rhizobia Symbiosis

**FIGURE 1:**
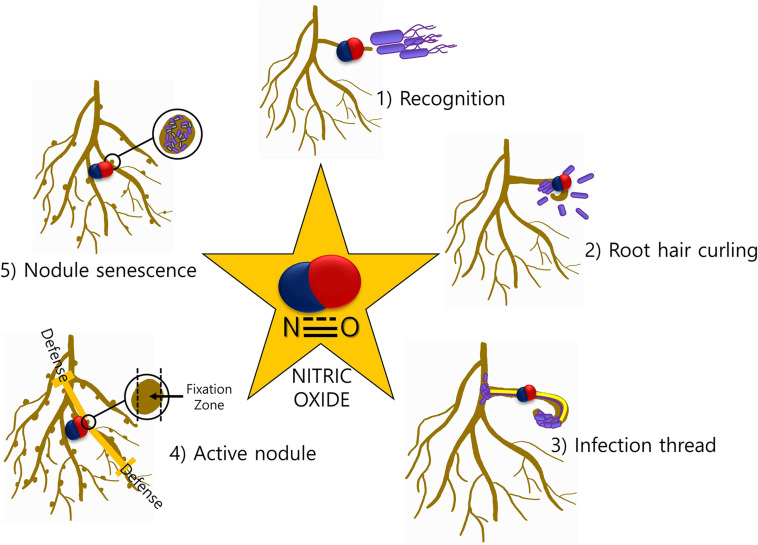
NO-mediated signaling during legume–rhizobia symbiosis. The presence of NO at various stages (1–5) of symbiosis is indicative of a major role of NO in mediating the communication between plants and microbes.

The involvement of NO in legume–rhizobia symbiosis has been thoroughly studied in the last decade. NO is involved at almost every step of the interaction between the legume host and rhizobia (Figure 1). Legume–rhizobium symbiosis is established following the recognition of rhizobia by the plants and the formation of root nodules that host the bacteria for nitrogen fixation. Nodule prevents the inhibition of nitrogenase activity by providing low oxygen environment. The process of nodulation begins with the production of bacterial nodulation factors (Nod-factors) in response to the chemical signals released by the root hairs. These Nod-factors trigger division in the roots and other responses downstream (Geurts and Bisseling, 2002). The growth of rhizobia induces root hair curling and the formation of infection thread which is a tubular and intracellular structure. Plant hormones cytokinin and auxin induce the formation of infection thread (Roy et al., 2020). The reproduction of rhizobia allows the continuous growth of rhizobia from where it reaches to the nodule primordium after reaching the base of the root hair cells (Geurts and Bisseling, 2002). Thereafter, the bacterial cells are released into the parenchyma cells of the nodule and the differentiation of bacterial cells to bacteroids begins. At this stage, morphological and physiological changes are required for this transformation. In the entire nodulation process, the involvement of NO is well documented. NO was reported to be induced at early stages (4 h post inoculation) but not at 10 and 24 h of plant–rhizobia interactions in *Lotus japonicus–Mesorhizobium loti* and *Medicago sativa–Ensifer meliloti* suggesting its role in signal recognition (Nagata et al., 2008). Similar results about NO induction and involvement during symbiosis establishment were observed in *Medicago truncatula–E. meliloti* symbiosis by another group (Del Giudice et al., 2011). Additionally, they have also reported NO detection during curling of root hair and formation of infection thread 2 and 6 days post-infection, respectively. Furthermore, scavenging the endogenously produced NO in the infection thread delays the nodule development.

## Role of Silicon in Plant–Microbe Interaction

Significant quantity of silicon present in the soil makes it the second most abundant element on the earth’s crust, and hence studies were carried out to understand its role in the rhizosphere. It was discovered that silicon could not only increase the biomass production but also had role in enhancing tolerance to biotic and abiotic stresses, thus supporting plants with stability and protection (Luyckx et al., 2017; López-Pérez et al., 2018). Interestingly, a study also discovered silicon uptake transporters from some legumes like soybean which were similar to the silicon transporters in hyper accumulating grasses (Deshmukh et al., 2013). The abundance of silicon in the rhizosphere and its role in plants lead to the investigations on the role of silicon in root nodulation and nitrogen fixation which started about 21 years back. The first study in *Vigna unguiculate* reported that silicon significantly affected root nodulation and nitrogen fixation depending upon the concentration of silicon, its forms (metasilicic acid or silicic acid), and growing substrate (liquid culture or sand culture) (Nelwamondo and Dakora, 1999). Later on, the same group reported that other than affecting nodule numbers and nitrogen fixation, silicon also affected the internal structure of the nodule. An increase in the size of peribacteroid space and cell wall thickness at high concentrations was reported along with an increase in the number of bacteroids and symbiosomes (Nelwamondo et al., 2001). A characteristic feature of silicon integration in most of the high silicon accumulating plants is an increase in cell wall thickness (Kumar et al., 2017). Interestingly, reports of the presence of silicon in the root nodules were also observed in *Cajanus cajan* (pigeonpea) genotypes (Garg and Singh, 2018). Nodule activity was promoted in two different genotypes of pigeonpea through the production of leghemoglobin. Another study in *M. sativa* also demonstrated the positive impact of silicon on root nodulation and also found it to be involved in biosynthesis of foliar amino acids (Johnson et al., 2017). Overall, it is clear that silicon employs beneficial impact on the symbiotic traits.

**FIGURE 2:**
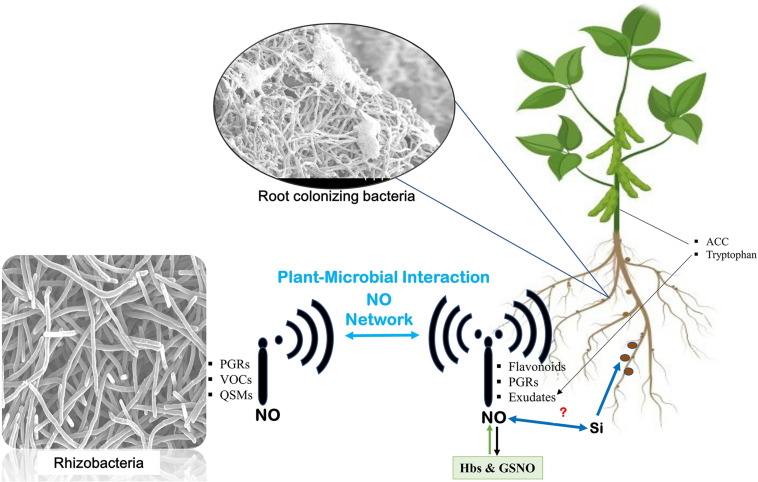
Nitric oxide network underground. Communication is established between plants and microbes through signals transmitted by microbes [such as plant growth regulators (PGRs), volatile organic compounds (VOCs), and quorum sensing molecules (QSMs) from microbes and similar signal transmission from host plants]. The signals from the host include flavonoids, PGRs, and exudates from the roots and aerial parts of the plant like 1-aminocyclopropane-1-carboxylic acid (ACC) and tryptophan. At every step, nitric oxide mediates the signaling and helps establish their interaction. Role of silicon in enhancing nitrogen fixation also leaves speculations about its relation with NO in mediating the signaling events.

Speculations about the joint effects of silicon and NO have been suggested recently. Combination of silicon and NO application in maize plants under cadmium stress is reported to increase plant growth and reduce cadmium uptake, accumulation, translocation, and bioaccumulation factors (Liu et al., 2020). However, the combined effect of silicon and NO in promoting root–microbiome interaction remains to be elucidated (Figure 2).

## Conclusion and Future Prospects

Rhizosphere is the area around a plant root inhabited by a diverse population of microorganisms; however, only the unique population can influence or can be influenced by the chemical signals released by either of them. Recent research has shown that NO participates in early basal signaling during plant–bacterial interactions. Bacteria and plants share several features of NO-producing pathways which suggests that NO acts as a signaling molecule during plant–microbe association.

Nitric oxide has been thoroughly studied as a signaling molecule in the last decade. NO may be produced endogenously by plant cells or may be generated as a result of activity of soil microorganisms. Soils are an important source of NO, and the presence of inorganic fertilizers can affect NO emissions into the environment. Since NO is a direct intermediate of nitrification and denitrification processes carried out by microorganisms during nitrogen fixation (Payne, 1981; Skiba and Smith, 1993; Feelisch and Martin, 1995), it suggests the possible contribution of roots and microbes in NO production and influencing the synthesis of NO on each other during symbiotic association (Molina-Favero et al., 2007). Furthermore, research needs to be conducted in understanding the role of silicon in plant–microbe signaling along with its combined effect with NO in mediating the microbial communication underground.

## Author Contributions

AP and B-GM have contributed equally in conceiving the idea and structure of the manuscript, and writing the first draft of the manuscript. D-SL illustrated the figures. G-ML contributed to the writing of the Section “NO in Plants and Bacteria.” MK contributed to the Section “Plant–Microbe Interaction: NO and the Plant Innate Immune System.” AH revised and improved the manuscript and figures. B-WY revised the whole manuscript. All authors contributed to the article and approved the submitted version.

## Conflict of Interest

The authors declare that the research was conducted in the absence of any commercial or financial relationships that could be construed as a potential conflict of interest.
